# Risk factors and clinical outcomes of arrhythmias in the medical intensive care unit

**DOI:** 10.1186/s40560-016-0131-x

**Published:** 2016-01-22

**Authors:** Rodrigo J. Valderrábano, Alejandro Blanco, Eduardo J. Santiago-Rodriguez, Christine Miranda, José Rivera-del Rio del Rio, Juan Ruiz, Robert Hunter

**Affiliations:** Endocrinology Department, Stanford University, Stanford, CA USA; Medicine Department, Universidad Central del Caribe School of Medicine, Bayamón, Puerto Rico; Retrovirus Research Center, Universidad Central del Caribe School of Medicine, Bayamón, Puerto Rico; Department of Medicine, 300 Pasteur Drive, Grant Building, Rm S025, Stanford, CA 94305-5103 USA

## Abstract

**Background:**

The clinical impact of arrhythmias on the continuum of critical illness is unclear, and data in medical intensive care units (ICU) is lacking. In this study, we distinguish between different types of arrhythmias and evaluate if their distinction is of clinical importance based on ICU length of stay and mortality outcomes.

**Methods:**

We performed a retrospective analysis of 215 patients in a community-based teaching hospital medical ICU. Variables gathered include sociodemographic data, arrhythmias identified and interpreted by the study team, and admission diagnoses coded into clinical mediator categories based on theorized common risk pathways. Univariable and multivariable Poisson regression models were used to identify risk factors for developing arrhythmias by type, prolonged length of stay, and hospital mortality.

**Results:**

Significant arrhythmia was detected in 28.8 % of subjects with most new arrhythmia events developing within the first 3 days of ICU stay. Acute myocardial ischemia and acute kidney injury at the time of ICU admission were associated with an increased risk of developing supraventricular arrhythmias (SVA) (RR = 2.02; 95 % CI 1.08–3.78 and RR = 1.93; 95 %CI 1.09–3.37, respectively). SVA in the first 3 days of ICU stay was associated with an increased risk of prolonged ICU stay (RR = 1.47; 95 % CI 1.09–1.97). After controlling for clinical mediators, development of SVA was not independently associated with in-hospital mortality. No mediators significantly increased the risk of developing ventricular arrhythmias (VA). VA were not associated to prolonged ICU stay but were associated with increased risk of hospital mortality (RR = 1.93; 95 % CI 1.18–3.15).

**Conclusions:**

It is important to distinguish between supraventricular and ventricular arrhythmias for outcomes in the medical ICU setting. Developing a new VA increases the risk of in-hospital mortality independently. Developing a new SVA increases the risk of having a prolonged ICU stay but does not appear to increase in-hospital mortality independently. These findings suggest that the development of a VA should be considered an independent morbid event and not necessarily the end result of a complicated clinical course, while a new SVA may be considered a cardiac complication of the disease continuum which may add complexity to an ICU stay.

## Background

Arrhythmias are common clinical events in the intensive care unit (ICU) setting. The frequency and prognosis associated with arrhythmias vary according to the clinical setting in which they occur. Arrhythmias have been reported to occur in 15.7 and 19.7 % of patients in the surgical and medical ICU settings, respectively [1]. In one epidemiologic study of critically ill patients, it was reported to be as high as 78 % [2]. Nevertheless, the clinical impact of many arrhythmias in the continuum of critical illness is somewhat unclear. In some studies, arrhythmias have been implicated as a marker of disease severity in critically ill patients [3]. They have also been associated with increased length of stay in the ICU, worse clinical outcome, and increased mortality [4, 5]. In the setting of cardiovascular surgery, the development of post-operative atrial fibrillation is considered a significant source of morbidity [6]. The development of ventricular arrhythmias (VA), although less common, are considered to be relevant clinical events [7, 8].

The clinical course of patients receiving care in the medical ICU is usually complex in nature with the presence of multiple concomitant insults to target organs in the form of renal, cardiac, and pulmonary illness. The co-existence of these morbid conditions in ICU patients makes it difficult to establish an association between a specific arrhythmia, an individual clinical scenario, and ultimately patient outcomes. The increasing human and resource utilization in ICU patients with arrhythmias argues for a more detailed evaluation of the clinical impact of arrhythmias in the ICU setting [9].

The majority of published studies evaluating the prevalence and relevance of arrhythmias in critically ill patients focus on cardiac vs. non-cardiac surgical patients, while data in the setting of a general medical ICU is less abundant. Our study focused on patients admitted to a medical ICU and aimed to elucidate the effects of simultaneous dysfunction in multiple target organs and the clinical scenarios associated to the development of atrial or ventricular arrhythmias. We hypothesized that similar clinical scenarios would have common mediating effects on heart arrhythmias and sought to establish which of these clinical scenarios would increase the risk of arrhythmias along with their association to patient outcomes. In addition, our study evaluated the distinction between supraventricular arrhythmias (SVA) and VA and their clinical importance on patient outcomes.

## Methods

### Study design

After Institutional Review Board (IRB) approval from the Universidad Central del Caribe School of Medicine, we performed a retrospective and prospective evaluation of patient records from patients older than 21 years of age admitted to the medical ICU at the Hospital Universitario Ramón Ruiz Arnau in Bayamón, Puerto Rico, from January 1, 2010 to February 28, 2011. Waiver of informed consent was granted by the IRB. Records from all admissions to the 5-bed general ICU of the community-based teaching hospital were evaluated. ICU patients were either direct admission to the ICU or transfers from the general wards. Patients were considered to be positive for arrhythmia if they presented with a new arrhythmia in the first 10 days of ICU stay. Patients with chronic arrhythmias were only considered if a new and different arrhythmia was detected. Arrhythmias which directly triggered cardiac decompensation and advanced cardiac life support (ACLS) procedures were included in our analysis. Arrhythmias and other waveforms which were present during ACLS or chest compressions were not included.

Arrhythmias were identified by a data gathering team that included two board certified internists and a senior internal medicine resident and was instructed to record any non-sinus rhythm. All electrocardiograms, rhythm strips (routinely printed every 4 h), and code cart strips available in the records were analyzed for the first 10 days of ICU stay for every patient in the study. Arrhythmias were first interpreted by the data gathering team using all available information and then confirmed by a review team of two board certified internists and one board certified cardiologist. If there was a disagreement on the appropriate interpretation of arrhythmia, a majority ruling was accepted. Arrhythmias were interpreted according to established definitions.

A supraventricular event was defined as having at least one non-sinus rhythm of supraventricular origin, excluding sinus arrhythmia and premature supraventricular contractions (PSC). A ventricular event was defined as having at least one non-sinus rhythm of ventricular origin, excluding premature ventricular contractions (PVC). Arrhythmias were classified according to their nature into Isolated (if the arrhythmia consisted of only one event) and Recurrent (if the same arrhythmia recurred with a period of at least 2 h of sinus rhythm between events) and according to their duration into Short-lasting (all events lasted under 24 h), Prolonged (at least one event over 24 h), and Permanent (event persisted continuously throughout the hospital stay) for descriptive purposes. Recurrent arrhythmias were considered as one event for analysis.

Arrhythmias were managed according to established protocols by treating physicians. Hemodynamically unstable patients with ventricular arrhythmias were treated with electrical cardioversion or defibrillation depending on morphology. Stable patients with monomorphic ventricular tachycardia were treated with IV amiodarone bolus and subsequent infusions. Hemodynamically unstable patients with SVA were given immediate electrical cardioversion. Stable patients with SVA were given IV diltiazem boluses and subsequent infusions. Correction of potassium levels were performed as required.

### Acute diagnoses

The ICU admission diagnoses were tabulated within 24 h of ICU admission. Acute ICU admission diagnoses were classified into general categories termed clinical mediators, which were divided according to their theorized modifying effect on arrhythmias. These did not include chronic pre-existing conditions unless there was an acute exacerbation present. The following clinical mediator categories were utilized: *Acute Respiratory Failure* (ARF), *Acute Myocardial Ischemia* (ISC), *Shock* (SHK), *Hyperglycemia* (GLU), *Acute Kidney Injury* (AKI), *Local infection* (L-INF), *Systemic infections/inflammatory state* (S-INF) (including sepsis and systemic inflammatory response syndrome), *Acute central nervous system event* (CNS) (including stroke and seizures), *Gastrointestinal bleeding* (GIB), *Acid-base disorders* (ABD), *Electrolyte disturbance* (E-DB), *Decompensated Congestive Heart Failure* (DCHF), and *Acute intoxication* (TOX).

The number of clinical mediators within a given patient was tallied. Patients were grouped based on the number of clinical mediators present: 0–2, 3–4, and 5 or more. These categories were used in statistical analyses as a measure of complexity of critical illness.

### Statistical analyses

Descriptive statistics, such as medians and proportions, were used to describe the population. Sociodemographic characteristics, lifestyle risk behaviors, presence of clinical conditions, patients’ ICU length of stay, and in-hospital mortality were all evaluated according to the occurrence of arrhythmias using the Kruskal-Wallis test on continuous variables and chi-square or Fisher’s exact tests on categorical variables. Post hoc assessments were carried out to identify which groups differed in the bivariate analyses. Differences in continuous variables were measured using Dunn’s test while differences in proportions were evaluated calculating *z* tests and adjusting by the Bonferroni method.

Poisson working models with robust variance estimators [10] were employed to determine the factors independently associated with arrhythmias, ICU length of stay >5 days, and in-hospital mortality, as this procedure was found to be the most appropriate [11]. For ICU length of stay, only arrhythmias in the first 3 days of stay were utilized for calculating extended stay (over 5 days) in order to maintain temporality and therefore assess risk. Sociodemographic variables and categories based on the sum of diagnostic mediators were included in the final models if their association with dependent variables resulted in a *p* < 0.20 on previous univariable analyses. Results were reported as relative risks (RR) with their respective 95 % confidence intervals (95 % CI). Statistical significance was set at the 0.05 level, and all tests were two-sided. Stata/SE (Version 12.1, College Station, TX, USA) and IBM SPSS Statistics 20 were used to carry out the analyses.

## Results

### Study population

**Fig. 1:**
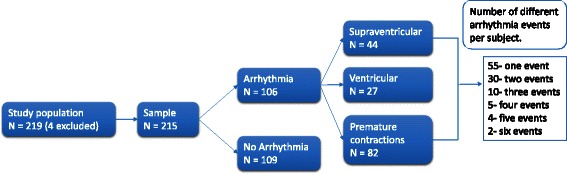
Arrhythmia flow chart by study subject. Flow of subjects enrolled into the study divided by the presence and type of the first arrhythmia. Subgroup of subjects with more than one type of arrhythmia is presented at the right.

A total of 267 patients were admitted to the medical ICU between January 1, 2010 and February 28, 2011. Two-hundred nineteen records were available for review, of which four were excluded due to age and missing data. Our cohort consisted of 215 subjects (Fig. 1). One hundred and six (49.3 % of total) patients had arrhythmia (rhythm other than sinus) with a total of 197 arrhythmias events. Fifty-five patients had one arrhythmia event, 30 had two events, 10 had three events, 5 had four events, 4 had five events, and 2 patients had six different arrhythmia events. Eighty-two patients had at least one premature contraction event (premature ventricular or supraventricular contractions), and in 44 of these patients premature events were the only arrhythmia event. A total of 62 (28.8 % of total) patients had supraventricular or ventricular arrhythmia events other than premature contractions. Of these, 44 patients had at least one SVA event, 27 patients had at least one VA event, 9 of these patients had at least one of both SVA and VA events. A total of 93 arrhythmia events were recorded. In Table 1, we present the descriptions of the arrhythmias documented. The most frequent supraventricular event was atrial fibrillation (33.3 % of SVA), which was also the most common arrhythmia overall (10.1 % of total events), while ventricular tachycardia was the most common ventricular event (51.5 % of VA).

**Table 1:** Description of the different types of arrhythmias detected in the ICU

Arrhythmia events	No. events	Events (%)	Total (%)
Supraventricular			
Atrial fibrillation	20	33.3	10.2
Junctional escape rhythm	13	21.7	6.6
Paroxysmal atrial tachycardia	8	13.3	4.1
Multifocal atrial tachycardia	6	10.0	3.0
Av node re-entry tachycardia	3	5.0	1.5
Atrial flutter	3	5.0	1.5
Accelerated junctional rhythm	3	5.0	1.5
Wandering pacemaker	2	3.3	1.0
Sinus pause/arrest	1	1.7	0.5
Mobitz type 1	1	1.7	0.5
Total supraventricular events	60	100.0	30.4
Premature supraventricular contractions (PSVC)^a^			
Premature atrial contractions	46	82.1	23.4
Atrial bigeminy	6	10.7	3.0
Premature junctional contractions	4	7.1	2.0
Total PSVC events	56	100.0	28.4
Ventricular			
Ventricular tachycardia	17	51.5	8.6
Ventricular escape rhythm	10	30.3	5.1
Ventricular fibrillation	2	6.1	1.0
Torsades de point	2	6.1	1.0
Accelerated ventricular rhythm	2	6.1	1.0
Total ventricular events	33	100.0	16.7
Premature ventricular contractions (PVC)^a^			
Premature ventricular	44	91.7	22.3
Ventricular bigeminy	4	8.3	2.0
Total PVC	48	100.0	24.4
Total arrhythmia events	197		100.0

^a^PSVC and PVC are presented separately but considered as one in the text

We analyzed the sociodemographic variables as a function of the absence of arrhythmias, and the presence of significant arrhythmias (SVA or VA), or premature contractions (PVC or PSC only) (Table 2). Patients with premature contractions were generally older and were more likely to be retired (51.2 %), have Medicare as their health insurance (38.6 %), have a history of alcohol drinking (38.1 %), and a history of hypertension (81.8 %) and congestive heart failure (22.7 %) than those in the other groups. Patients with arrhythmias had a significantly higher in-hospital mortality rate (46.8 %) when compared to the other two groups (no arrhythmias: 19.3 % and premature complexes: 15.9 %).

**Table 2:** Sociodemographic profile and selected clinical variables of participants according to the presence or absence of significant arrhythmia and presence of premature complexes

	All (*n* = 215)	No arrhythmia (*n* = 109)	Arrhythmia^a^ (*n* = 62)	Premature complexes^b^ (*n* = 44)	*p* value
Sociodemographic characteristics					
Age, median (IQR)	62 (48–77)	51 (39–64)^c,d^	66 (55–81)^c^	72 (64–82)^d^	<0.001
Male gender, *n* (%)	128 (59.5)	62 (56.9)	37 (59.7)	29 (65.9)	0.588
Race/ethnicity (*n* = 198), *n* (%)					
Hispanic white	133 (67.2)	62 (62.6)	39 (67.2)	32 (78.1)	0.265
Hispanic black	61 (30.8)	34 (34.3)	19 (32.8)	8 (19.5)	
Other	4 (2.0)	3 (3.0)	0 (0)	1 (2.4)	
Civil status (*n* = 201), *n* (%)					
Single/divorced	91 (45.3)	46 (46.9)	29 (49.2)	16 (36.4)	0.083
Married	79 (39.3)	43 (43.9)	17 (28.8)	19 (43.2)	
Widowed	31 (15.4)	9 (9.2)	13 (22.0)	9 (20.5)	
Education (*n* = 139), *n* (%)					
<High school	78 (56.1)	41 (55.4)	20 (60.6)	17 (53.1)	0.818
High school graduate or more	61 (43.9)	33 (44.6)	13 (39.4)	15 (46.9)	
Employment (*n* = 181), *n* (%)					
Employed	42 (23.2)	24 (25.3)	11 (24.4)	7 (17.1)	0.047
Unemployed	80 (44.2)	48 (50.5)	19 (42.2)	13 (31.7)	
Retired/disabled	59 (32.6)	23 (24.2)^c^	15 (33.3)	21 (51.2)^c^	
Medical insurance, *n* (%)					
Medicaid	135 (62.8)	80 (73.4)^c,d^	34 (54.8)^c^	21 (47.7)^d^	0.013
Medicare	46 (21.4)	14 (12.8)^c^	15 (24.2)	17 (38.6)^c^	
Private	19 (8.8)	8 (7.3)	7 (11.3)	4 (9.1)	
Uninsured	15 (7.0)	7 (6.4)	6 (9.7)	2 (4.6)	
Risky behaviors					
Tobacco smokers (*n* = 202), *n* (%)	78 (38.6)	36 (36.4)	25 (41.7)	17 (39.5)	0.793
Illicit drug users (*n* = 204), *n* (%)	38 (18.6)	24 (23.8)	9 (14.8)	5 (11.9)	0.164
Alcohol drinkers (*n* = 201), *n* (%)	47 (23.4)	19 (19.4)	12 (19.7)	16 (38.1)	0.040
Pre-existing conditions					
Hypertension (*n* = 210), *n* (%)	134 (63.8)	57 (53.3)^c^	41 (69.5)	36 (81.8)^c^	0.002
Diabetes (*n* = 211), *n* (%)	96 (45.5)	49 (45.8)	25 (41.7)	22 (50.0)	0.698
CHF (*n* = 209), *n* (%)	26 (12.4)	8 (7.6)^c^	8 (13.3)	10 (22.7)^c^	0.038
CAD (*n* = 209), *n* (%)	67 (32.1)	31 (29.5)	20 (33.3)	16 (36.4)	0.695
Dyslipidemia (*n* = 210), *n* (%)	35 (16.7)	14 (13.2)	11 (18.3)	10 (22.7)	0.333
HIV (*n* = 210), *n* (%)	11 (5.2)	5 (4.7)	3 (5.0)	3 (6.8)	0.848
CKD (*n* = 210), *n* (%)	27 (12.9)	11 (10.4)	8 (13.3)	8 (18.2)	0.426
Asthma/COPD (*n* = 210), *n* (%)	37 (17.6)	21 (19.8)	8 (13.3)	8 (18.2)	0.571
Liver disease (*n* = 212), *n* (%)	20 (9.4)	12 (11.1)	5 (8.3)	3 (6.8)	0.745
Outcomes					
Length of stay in ICU (days)					
Total	1964	968	686	310	–
Median (IQR)	5 (3–11)	5 (3–8)	7 (4–13)	4 (3–11)	0.089
In-hospital mortality, *n* (%)	57 (26.5)	21 (19.3)^c^	29 (46.8) ^c, d^	7 (15.9)^d^	<0.001

*p* values were computed using Kruskal-Wallis test on continuous variables and chi-square test or Fisher’s exact test on categorical variables. Post hoc evaluations were made using Dunn’s test and *z* tests with Bonferroni corrections

^a^Patients who presented supraventricular and/or ventricular arrhythmias

^b^Patients who presented premature atrial contractions (PACs) and/or premature ventricular complexes (PVCs) but no arrhythmias

^c,d^Denote which groups have statistically significant differences after post hoc comparisons (*p* < 0.05)

**Fig. 2:**
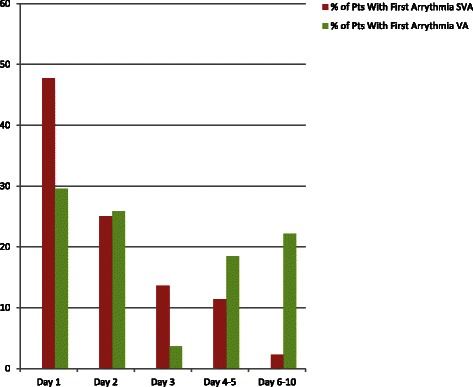
Onset of the first arrhythmia by ICU stay and type. Initial detection of the first arrhythmia for subjects admitted to the ICU, organized by day of ICU stay and site of origin of significant arrhythmia. Premature complexes were excluded from analysis

The majority (73.0 %) of patients presented their first event within 72 h of admission to the ICU (Fig. 2). Of the patients that had SVA events, 47.7 % had their first event on the ICU admission day, 25.0 % on ICU day 2, and 13.6 % on ICU day 3, with a cumulative 86.3 % in the first 72 h. Approximately one tenth of the first SVA events occurred on ICU days 4 and 5, and 2.3 % of events occurred on or after ICU day 6. Of the patients that had VA events, 29.6 % had their first event on the ICU admission day, 25.9 % on ICU day 2, and 3.7 % on ICU day 3, with a cumulative 59.2 % in the first 72 h. A total of 18.5 % had their first VA event on ICU days 4 and 5, and 22.2 % of events occurred on or after ICU day 6. Similar patterns emerged in patients which had premature contractions as their sole arrhythmia events. Of these patients, 47.7 % had their first event on the day of ICU admission, 22.7 % on ICU day 2, and 6.8 % on ICU day 3 with a cumulative 77.2 % on that day, while 11.4 % of the first premature events occurred on ICU days 4 and 5 and 11.3 % of events occurred on or after ICU day 6. Of the SVA events, 65.9 % were of short duration. Of the VA events, 96.3 % were of short duration.

We also analyzed the clinical mediators associated with the arrhythmias in the study (Table 3). The most common ICU clinical mediators were acute respiratory failure (50.2 %) followed by local infection (47.0 %) and acute kidney injury (42.3 %). There was a significant difference in the occurrence of acute kidney injury (*p* = 0.004), decompensated CHF (*p* = 0.016), and systemic infection/inflammation (*p* = 0.036) between the arrhythmia status groups. Patients with GI bleeding and intoxication were few in number, and they lacked the power to perform statistical analysis, so these mediators were excluded from regression models.

**Table 3:** Association of significant arrhythmia, premature complexes and no arrhythmias with clinical mediators responsible for ICU admission

	All (*n* = 215)	No arrhythmia (*n* = 109)	Arrhythmia^a^ (*n* = 62)	Premature complexes^b^ (*n* = 44)	*p* value
Acute respiratory failure, *n* (%)	108 (50.2)	47 (43.1)	37 (59.7)	24 (54.6)	0.093
Shock, *n* (%)	17 (7.9)	6 (5.5)	9 (14.5)	2 (4.6)	0.090
Acute Myocardial Ischemia, *n* (%)	45 (20.9)	19 (17.4)	16 (25.8)	10 (22.7)	0.410
Decompensated CHF, *n* (%)	30 (14.0)	11 (10.1)	7 (11.3)	12 (27.3)	0.016
Hyperglycemia, *n* (%)	86 (40.0)	45 (41.3)	25 (40.3)	16 (36.4)	0.852
Acute kidney injury, *n* (%)	91 (42.3)	35 (32.1)	36 (58.1)	20 (45.5)	0.004
Local infection, *n* (%)	101 (47.0)	45 (41.3)	34 (54.8)	22 (50.0)	0.210
Systemic infection, *n* (%)	60 (27.9)	25 (22.9)	25 (40.3)	10 (22.7)	0.036
Acute CNS event, *n* (%)	19 (8.8)	9 (8.3)	5 (8.1)	5 (11.4)	0.802
Gastrointestinal bleeding, *n* (%)	15 (7.0)	13 (11.9)	0 (0)	2 (4.6)	0.006
Acid/base disturbance, *n* (%)	40 (18.6)	21 (19.3)	11 (17.7)	8 (18.2)	0.967
Electrolyte imbalance, *n* (%)	32 (14.9)	16 (14.7)	7 (11.3)	9 (20.5)	0.425
Intoxication, *n* (%)	6 (2.8)	4 (3.7)	0 (0)	2 (4.6)	0.288
Mediators, *n* (%)					
0–2	84 (39.1)	50 (45.9)	19 (30.7)	15 (34.1)	0.054
3–4	92 (42.8)	47 (43.1)	28 (45.2)	17 (38.6)	
≥5	39 (18.1)	12 (11.0)	15 (24.2)	12 (27.3)	

*p* values were computed chi-square test or Fisher’s exact test

^a^Patients who presented supraventricular and/or ventricular arrhythmias

^b^Patients who presented premature atrial contractions (PACs) and/or premature ventricular complexes (PVCs) but no arrhythmias

Results of univariable and multivariable regression analyses evaluating the association of clinical mediators, sex, and age with the different types of arrhythmias are presented in Table 4. Older age, acute respiratory failure, shock, acute myocardial ischemia, acute kidney injury, local infection, and systemic infection were found to be associated to SVAs at a level of *p* <0 .20. When these factors were included in the multivariable model, only acute myocardial ischemia (RR = 2.02; 95 %CI 1.08–3.78) and acute kidney injury (RR = 1.93; 95 % CI 1.09–3.37) were associated with a significant increased risk of SVAs. No clinical mediators were identified which could be associated with the presence of VAs. Older age categories and decompensated congestive heart failure were found to be associated to premature complexes in univariable analyses. The multivariable model revealed older ages (51–70 years and over 71 years vs. 21–50 years) were associated with an increased risk of premature complexes (RR = 4.82; 95 % CI 1.48–15.70 and RR = 6.07; 95 % CI 1.88–19.65, respectively).

**Table 4:** Univariable and multivariable analysis of the association between age, sex, and clinical mediators associated with the presence of significant ventricular, supraventricular arrhythmia, and premature complexes

		Supraventricular (*n* = 44)				Ventricular (*n* = 27)				Premature complexes (*n* = 82)			
		Univariable		Multivariable^a^		Univariable		Multivariable^b^		Univariable		Multivariable^a^	
Variable	*n* (%)	RR (95 % CI)	*p* value	RR (95 % CI)	*p* value	RR (95 % CI)	*p* value	RR (95 % CI)	*p* value	RR (95 % CI)	*p* value	RR (95 % CI)	*p* value
Sex													
Female	87 (40.5)	Ref				Ref				Ref			
Male	128 (59.5)	0.98 (0.57–1.68)	0.946	–	–	0.99 (0.48–2.03)	0.975	–	–	1.42 (0.73–2.77)	0.301	–	–
Age													
21–50	66 (30.7)	Ref		Ref		Ref				Ref		Ref	
51–70	79 (36.7)	1.78 (0.82–3.86)	0.147	1.26 (0.56–2.82)	0.572	1.53 (0.60–3.93)	0.375	–	–	5.29 (1.63–17.15)	0.005	4.82 (1.48–15.70)	0.009
>71	70 (32.6)	2.24 (1.05–4.77)	0.037	1.47 (0.68–3.14)	0.325	1.57 (0.60–4.09)	0.355	–	–	6.91 (2.17–22.08)	0.001	6.07 (1.88–19.65)	0.003
ARF	108 (50.2)	1.73 (0.99–3.02)	0.052	1.47 (0.86–2.49)	0.151	1.07 (0.53–2.17)	0.858	–	–	1.19 (0.70–2.02)	0.523	–	–
SHK	17 (7.9)	2.20 (1.16–4.18)	0.015	1.62 (0.74–3.54)	0.227	2.03 (0.79–5.19)	0.142	–	–	0.55 (0.15–2.10)	0.386	–	–
ISC	45 (20.9)	1.58 (0.91–2.77)	0.107	2.02 (1.08–3.78)	0.027	0.66 (0.24–1.81)	0.416	–	–	1.11 (0.59–2.08)	0.741	–	–
AKI	91 (42.3)	2.38 (1.37–4.14)	0.002	1.93 (1.09–3.37)	0.022	1.47 (0.72–2.97)	0.287	–	–	1.14 (0.67–1.93)	0.638	–	–
DCHF	30 (14.0)	0.79 (0.34–1.85)	0.588	–	–	1.07 (0.40–2.89)	0.890	–	–	2.31 (1.35–3.97)	0.002	1.66 (0.97–2.84)	0.066
GLU	86 (40.0)	1.37 (0.81–2.32)	0.241	–	–	0.63 (0.29–1.38)	0.249	–	–	0.86 (0.49–1.49)	0.584	–	–
L-INF	101 (47.0)	1.63 (0.95–2.80)	0.076	1.08 (0.58–2.00)	0.815	1.05 (0.52–2.12)	0.896	–	–	1.13 (0.67–1.91)	0.653	–	–
S-INF	60 (27.9)	1.96 (1.17–3.30)	0.011	1.67 (0.88–3.16)	0.116	1.52 (0.74–3.13)	0.257	–	–	0.76 (0.40–1.44)	0.401	–	–
ABD	40 (18.6)	0.83 (0.40–1.72)	0.613	–	–	1.25 (0.54–2.90)	0.603	–	–	0.97 (0.49–1.93)	0.936	–	–
E-DB	32 (14.9)	0.90 (0.42–1.96)	0.797	–	–	0.46 (0.11–1.84)	0.271	–	–	1.47 (0.78–2.76)	0.231	–	–
CNS	19 (8.8)	0.75 (0.26–2.21)	0.608	–	–	0.83 (0.21–3.23)	0.783	–	–	1.32 (0.59–2.96)	0.496	–	–

*ARF* acute respiratory failure, *SHK* shock, *ISC* acute myocardial ischemia, *AKI* acute kidney injury, *DCHF* decompensated CHF, *GLU* hyperglycemia, *L-INF* local infection, *S-INF* systemic infection, *ABD* acid/base disturbance, *E-DB* electrolyte imbalance, *CNS* acute CNS event

^a^Factors included in the model had *p* < 0.20 in univariable analyses

^b^Model was not built because only one factor had a *p* < 0.20

### Outcomes

Chi-square analysis revealed that prolonged or permanent duration of SVAs was associated with increased ICU length of stay (more than 5 days) (*p* = 0.015). Prolonged or permanent duration of VA events was not associated to increased ICU stay (*p* = 0.437). Prolonged or permanent duration of SVA and VA events was not associated to in-hospital mortality, (*p* = 0.759 and *p* = 0.999, respectively).

Results of univariable and multivariable analyses of the associations between arrhythmias and length of ICU stay (over 5 days) and in-hospital mortality are shown in Table 5. Older age, having three or more clinical mediators, and presenting SVA in the first 3 days of ICU stay were associated with a prolonged ICU stay (more than 5 days) at a *p* < 0.20. Notably, having VAs or premature complexes in the first 3 days of ICU stay was not associated with prolonged ICU stay in univariable analyses. The multivariable model confirmed the association of SVA in the first 3 days of ICU stay with an increased risk of prolonged ICU stay (RR = 1.47; 95 % CI 1.09–1.97).

**Table 5:** Univariable and multivariable analysis of the risk of prolonged ICU stay and in-hospital mortality after development of a new significant ventricular, supraventricular arrhythmia, and premature complexes

			Prolonged ICU stay^a,b^				Mortality			
			Univariable		Multivariable^c^		Univariable		Multivariable^c^	
	*n* (%)		RR (95 % CI)	*p* value	RR (95 % CI)	*p* value	RR (95 % CI)	*p* value	RR (95 % CI)	*p* value
Sex										
Female	87 (40.5)		Ref				Ref			
Male	128 (59.5)		1.02 (0.76-1.37)	0.897	–	–	0.87 (0.56–1.36)	0.542	–	–
Age										
21–50	66 (30.7)		Ref				Ref		Ref	
51–70	79 (36.7)		1.67 (1.13–2.48)	0.011	1.60 (1.09–2.36)	0.017	1.67 (0.84–3.32)	0.143	1.32 (0.68–2.55)	0.411
>71	70 (32.6)		1.46 (0.96–2.21)	0.078	1.34 (0.87–2.05)	0.185	2.55 (1.34–4.85)	0.004	1.76 (0.91–3.38)	0.092
Mediators										
0–2	84 (39.1)		Ref		Ref		Ref		Ref	
3–4	92 (42.8)		1.44 (1.03–2.03)	0.034	1.35 (0.97–1.89)	0.075	4.30 (2.01–9.22)	<0.001	3.98 (1.89–8.37)	<0.001
≥5	39 (18.1)		1.39 (0.92–2.11)	0.121	1.17 (0.76–1.80)	0.480	5.23 (2.36–11.59)	<0.001	4.12 (1.80–9.43)	0.001
Arrhythmias										
SVA	38 (17.7)^b^	44 (20.5)	1.55 (1.17–2.07)	0.003	1.47 (1.09–1.97)	0.011	1.94 (1.25–3.02)	0.003	1.38 (0.89–2.15)	0.146
VA	16 (7.4)^b^	27 (12.6)	0.94 (0.53–1.67)	0.822	–	–	2.06 (1.29–3.29)	0.003	1.93 (1.18–3.15)	0.008
PC	59 (27.4)^b^	82 (38.1)	0.83 (0.59–1.18)	0.310	–	–	1.36 (0.87–2.12)	0.174	0.94 (0.61–1.45)	0.789

*SVA* supraventricular arrhythmias, *VA* ventricular arrhythmias, *PC* premature complexes

^a^ICU stay of more than 5 days

^b^Arrhythmias considered for this analysis occurred during the first 3 days at ICU

^c^Factors included in the model had *p* < 0.20 in univariable analyses

Older age, higher number of clinical mediators, SVA, VA, and premature complexes were all associated with an increased in-hospital mortality at *p* < 0.20. The multivariable Poisson regression model suggested a significant association of VA and increased risk of in-hospital mortality (RR = 1.93; 95 % CI 1.18–3.15), while controlling for the other factors. Finally, SVA and premature complexes were not associated with mortality.

## Discussion

In our single-center medical ICU study, we found that the incidence of significant arrhythmias (SVA or VA) was 28.8 % and that supraventricular arrhythmias were associated with a 47 % increased risk of prolonged ICU length of stay while ventricular arrhythmias were associated with a 93 % increased risk of in-hospital mortality.

We found that most new arrhythmias occurred in the first 3 days of ICU stay. After adjusting for methodological differences (our ICU day 1 = post op day 0), this pattern is comparable to several studies that have shown arrhythmias shortly after thoracic surgery [7, 12, 13]. Other studies in medical ICUs have shown similar patterns [14]. This suggests that the ICU admission event may be considered comparable to surgery in terms of the temporality of developing new arrhythmias.

One objective of our study was aimed at analyzing acute conditions which may modulate development of arrhythmias. We found that having acute myocardial ischemia and acute kidney injury upon admission to the ICU was associated with increased risk of developing SVA after controlling for other factors. This is in line with data where acute infarction was associated with rhythm abnormalities [15] and new onset atrial fibrillation [16]. Increases in cardiac dysrhythmias have also been found to be in greater prevalence in complex cardiovascular patients [17]. Elevated initial serum creatinine levels have also been associated to the development of atrial fibrillation, a type of SVA [16]. Other studies have also found association between atrial fibrillation and acute kidney injury in cardiac surgery patients [18]. Our findings suggest that acute myocardial ischemia and acute kidney injury may be considered independent risk factors for developing SVA when present at ICU admission.

Our results indicate that development of VAs was not found to be associated to any particular diagnostic mediator. We also found that VAs occurred mostly in the first 72 h after admission to the ICU (>50 %), and there was a trend to continue developing new arrhythmias throughout the entirety of ICU stay more than SVAs. This may mean that ICU admission diagnoses are not good predictors of developing VAs and that the developing clinical condition throughout an ICU stay is more important than the admission diagnosis for ventricular arrhythmias. Indeed, other studies have shown that chronic conditions are not good predictors of new onset sustained VA in coronary artery bypass graft patients [7, 19]. An alternate hypothesis for the lack of associations in VA could be that patients who developed ventricular arrhythmias had different predisposing baseline conditions. To test this, we performed univariable analyses of pre-existing conditions (results not presented), and there were no significant differences between our comparison group and the groups with different types of arrhythmias. So, in our cohort, the lack of associations cannot be explained by this hypothesis.

Supraventricular arrhythmias were significantly associated with mortality in univariable analysis, but this effect was attenuated when included in the regression model with the clinical mediators derived from ICU admission diagnoses, supporting previous data that suggests that SVA are markers of disease severity [12, 14], although, in certain clinical scenarios, arrhythmias such as atrial fibrillation can add significant morbidity [20, 21]. Indeed, SVAs were found to be associated with prolonged length of stay when controlled for age and diagnostic mediators, replicating the pattern that has been demonstrated in other studies [4, 22]. We found that SVA events of over 24 h duration were also associated to prolonged length of ICU stay, supporting the notion that development of an atrial arrhythmia adds to the complexity of managing an ICU patient, which may increase ICU length of stay.

Ventricular arrhythmias were not associated with prolonged length of stay but were associated with in-hospital mortality. Although we did not directly measure the temporal relationship between mortality and VAs, one explanation could be that patients with higher mortality have shorter ICU length of stay than survivors. The association with mortality persisted after controlling for clinical mediators and other types of arrhythmias. Numerous mediators in a single patient may represent increased complexity of illness overall, and while increasing number of clinical mediators increased the risk of in-hospital mortality, the association of VA and mortality was not significantly attenuated by the inclusion of this variable in our analyses. One study on the mode of death after cardiac arrest revealed that almost 75 % of patients that died after in-hospital cardiac arrest due to ventricular tachycardia, ventricular fibrillation, or pulseless rhythm were not due to purely cardiovascular causes [23]. Our data suggests that ventricular arrhythmias in the medical ICU patient population may be considered an independent contributor to mortality instead of part of the continuum of critical illness or the end result of a complicated clinical course.

We found that decompensated congestive heart failure and older age increased the risk of developing premature complexes. Premature ventricular complexes are common and may increase the risk of other ventricular dysrhythmias [24] and cardiomyopathy [25]. In our study, the development of PVCs and PSCs were not associated with increased risk of prolonged ICU length of stay or mortality. The subset of patients who had premature complexes as their only arrhythmia in our cohort were older and more frequently had congestive heart failure and hypertension as pre-existing conditions. This suggests that the development of these abnormal rhythms may be a marker of baseline heart disease, but not an important contributing event in the evolution of an ICU hospitalization.

Our study continues to add to the currently limited data on arrhythmias in medical ICUs. It expands on previous reports by detailing what clinical scenarios are likely to increase the risk of different types of arrhythmias and by exploring the differential effect of SVA vs. VA on outcomes. Our study is limited by a relatively small sample size. Our sample was also obtained from a pool of patients from a purely medical ICU, so that patients with significant surgical disease such as severe valvular heart disease were not represented.

## Conclusions

Our findings suggest that arrhythmias behave similarly in medical patients compared to surgical patients in terms of the temporality of developing new arrhythmias. Our findings also suggest that acute myocardial ischemia and acute kidney injury are related to development of SVAs, that premature complexes do not add to morbidity or mortality, that SVAs add to morbidity but not mortality, and that VAs are independent risk factors for mortality.

## Abbreviations

ABD: acid base disorder
ACLS: advanced cardiovascular life support
AKI: acute kidney injury
ARF: acute respiratory failure
CI: confidence intervals
CNS: acute central nervous system event
DCHF: decompensated congestive heart failure
E-DB: electrolyte disturbance
GIB: gastrointestinal bleed
GLU: hyperglycemia
ICU: intensive care unit
ISC: acute myocardial ischemia
L-INF: local infection
PSC: premature supraventricular contraction
PVC: premature ventricular contraction
RR: relative risk
SHK: shock
S-INF: systemic infection
SVA: supraventricular arrhythmia
TOX: acute toxicity
VA: ventricular arrhythmia
